# Evaluation of chemical chaperones based on the monitoring of Bip promoter activity and visualization of extracellular vesicles by real‐time bioluminescence imaging

**DOI:** 10.1002/bio.3388

**Published:** 2017-09-20

**Authors:** Tomohisa Horibe, Nanako Okushima, Aya Torisawa, Ryutaro Akiyoshi, Yoko Hatta‐Ohashi, Hirobumi Suzuki, Koji Kawakami

**Affiliations:** ^1^ Department of Pharmacoepidemiology, Graduate School of Medicine and Public Health Kyoto University Kyoto Japan; ^2^ Evaluation Technology Department 1 Olympus Corporation Hachioji‐shi Tokyo Japan

**Keywords:** bioluminescence, Bip/GRP78 promoter activity, chemical chaperone, extracellular vesicles, imaging

## Abstract

It is known that endoplasmic reticulum (ER) stress in cells and extracellular vesicles (EVs) plays a significant role in cancer cells, therefore the evaluation of compounds that can regulate ER stress and EV secretion would be a suitable system for further screening and development of new drugs. In this study, we evaluated chemical chaperones derived from natural products based on monitoring Bip/GRP78 promoter activity during cancer cell growth, at the level of the single cell, by a bioluminescence microscopy system that had several advantages compared with fluorescence imaging. It was found that several chemical chaperones, such as ferulic acid (FA), silybin, and rutin, affected the activity. We visualized EVs from cancer cells using bioluminescence imaging and showed that several EVs could be observed when using CD63 fused with NanoLuc luciferase, which has a much smaller molecular weight and higher intensity than conventional firefly luciferase. We then examined the effects of the chemical chaperones on EVs from cancer cells by bioluminescence imaging and quantified the expression of CD63 in these EVs. It was found that the chemical chaperones examined in this study affected CD63 levels in EVs. These results showed that imaging at the level of the single cell using bioluminescence is a powerful tool and could be used to evaluate chemical chaperones and EVs from cancer cells. This approach may produce new information in this field when taken together with conventional and classical methods.

**Abbreviations used**
CCD: charge coupled device
ER: endoplasmic reticulum
EV: extracellular vesicles
FA: ferulic acid
FACS: fluorescence activated cell sorter
FBS: fetal bovine serum
GFP: green fluorescent protein
HD: hard disc
PBS: phosphate‐buffered saline
ROI: regions of interests
SD: standard deviation
TEM: transmission electron microscopy

## 1 INTRODUCTION

A quality control system for newly synthesized cellular proteins is essential and indispensable for all prokaryotes and eukaryotes.^1^ Molecular chaperones play significant roles in this system such as distinguishing between unfolded and folded newly synthesized proteins and assisting in their correct folding. Correct folding is an indispensable step in the intrinsic function of these proteins, especially in the regulation of protein homeostasis, including influencing cell expression levels when responding to environmental and physiological stresses such as heat shock or ER stress.^1^, ^2^, ^3^, ^4^ Several low‐molecular‐weight compounds derived from natural products, such as ferulic acid (*trans*‐4‐hydroxy‐3‐methoxycinnamic acid; FA), sodium 4‐phenylbutyrate (4PBA), γ‐oryzanol, silybin, kojic acid (5‐hydroxy‐2‐hydroxymethyl‐4‐pyrone), and rutin (quercetin 3‐rutinoside) are known to have chaperone‐like activities, can function as chemical chaperones or antioxidants, and are able to regulate chaperone proteins or ER stress.^5^, ^6^, ^7^, ^8^, ^9^, ^10^, ^11^ It is known that binding immunoglobulin protein, Bip (also known as GRP78) is a molecular chaperone protein mainly located in ER^4^ and is often used as marker when examining ER stress, during which Bip/GRP78 is upregulated in cells.^5^, ^9^ Thus, Bip/GRP78 is also used to examine the effect of chemical chaperones on ER stress in cells.^5^, ^9^ Several of these chemical chaperones are currently expected to be used in the clinic, as 4PBA has been approved by the United States Food and Drug Administration (US FDA) for the treatment of urea cycle disorders.^7^ Therefore, a method to evaluate the effects of these chemical chaperones would be useful not only for screening for new chemical chaperones but also for the development of new drugs, using these chemical chaperones as lead compounds.

Extracellular vesicles including exosomes have been widely investigated and are receiving increasing attention in studies on their basic function and content to their development as therapeutic or diagnostic tools for several diseases.^12^ They consist of a lipid bilayer membrane and contain proteins, receptors, nucleic acid including microRNAs, chemicals, and structural contents derived from the original cell.^13^, ^14^, ^15^ Extracellular vesicles are usually classified according to their size and biological specificity as exosomes (30–100 nm),^13^ microvesicles (50–1000 nm)^15^ and apoptotic bodies (500–2000 nm).^15^ Exosomes are currently the most well studied type of EV and studies have mainly focussed on cancer cell biology, treatment and diagnosis.^12^ In addition, it has been reported recently that the ER stress response could affect EV secretion or content.^16^, ^17^ Therefore a method for the detection and real‐time imaging of EVs, including exosomes, would be useful not only to investigate EV mechanisms but also to evaluate drugs that regulate EVs that are secreted from cancer cells.

Previously, we have monitored Bip/GRP78 promoter activity during cancer cell growth by real‐time imaging at the level of the single cell using a bioluminescence microscopy system, in which a cooling charge coupled device (CCD) camera was successfully combined with a microscope as a new optical system.^18^ Bioluminescence and morphology was detected and observed at the single cell level using this system, and was important in selecting live cells and excluding false positives. This technique is a suitable and available tool not only for gene analysis related to the regulation of unfolded protein responses in cancer cells but also for evaluation of anti‐cancer agent efficacy.^18^ The main advantages in using bioluminescence imaging from luciferin–luciferase reaction emissions are its lower background, and higher quantification.^19^, ^20^ In addition there is low damage to living cells compared with the use of fluorescent proteins or fluorescein isothiocyanate, as an excitation light source is not needed for detection.^19^, ^20^ Monitoring by real‐time imaging using bioluminescence would therefore provide additional information when combined with conventional and classic assays.

It is known that ER stress and EVs play significant roles in cancer cells,^4^, ^12^ therefore compounds that can regulate ER stress, EV secretion and EV protein content might be new candidates as available drugs for new types of cancer treatment. The evaluation of such new compounds would be useful for new drug screening, discovery, and development. In this study, we evaluated several chemical chaperones based on Bip/GRP78 promoter activity monitoring during cancer cell growth using real‐time bioluminescence imaging at the single cell level and using bioluminescence microscopy. Extracellular vesicles, including exosomes, secreted from cancer cells were evaluated using bioluminescence emitted from NanoLuc,^21^ the intensity of which was much higher than that of conventional firefly luciferase. CD63 levels in these EVs were quantified in the presence or absence of chemical chaperones. Two kinds of luciferases were observed simultaneously to evaluate imaging using a bioluminescence microscopy system.

## 2 EXPERIMENTAL

### 2.1 Materials

FA and γ‐oryzanol were purchased from Tokyo Chemical, Inc. (Tokyo, Japan). 4PBA was purchased from LKT Laboratory, Inc. (St. Paul, MN, USA). Silybin was purchased from Cayman Chemical (Ann Arbor, MI, USA). Kojic acid and rutin were purchased from WAKO (Osaka, Japan). The human glioblastoma U251 cell line was obtained as reported previously.^22^ U251 cells stably transfected with pBipPro‐Luc (U251/Luc), in which the promoter region of Bip was cloned into the pGL4.14 vector (Promega, Madison, WI, USA), were established previously.^18^ The cells were cultured in RPMI‐1640 medium containing 10% fetal bovine serum (FBS) as reported previously.^22^ Other reagents were mostly from Nacalai Tesque (Kyoto, Japan).

### 2.2 Construction of reporter plasmid and expression vector, transfection, and establishment of stable cell lines

The pBipPro‐Luc reporter plasmid was constructed as reported previously.^22^ The CD63 expression vector fused with NanoLuc (CD63NLuc) was constructed using pNLF1C (Promega), in which artificially synthesized human CD63 cDNA (ThermoFisher, Waltham, MA, USA) with the stop codon replaced with GGC (glycine) was cloned into the multiple‐cloning site of the vector. Transient transfection with the constructed vectors was performed using Lipofectamine LTX (Invitrogen, Carlsbad, CA, USA) according to the manufacturer's protocol. U251 cells stably transfected with Mock, pNLF1C alone, or CD63NLuc (Mock/U251 or CD63NLuc/U251) were established through the selection in cell culture medium containing hygromycin B (Nacalai Tesque) as described previously.^18^

### 2.3 Imaging and time‐course analysis using bioluminescence

Imaging using bioluminescence at the single cell level was performed using the bioluminescence microscopy system LUMINOVIEW (LV200) Imaging System (Olympus, Tokyo, Japan) as reported previously.^22^ Briefly, a culture dish was kept at 37°C during the observation, and images were obtained using a × 100 magnification objective lens after addition of d‐luciferin (Promega) to the culture medium at the final concentration of 500 μM. The Nano‐Glo Live Cell Assay System (Promega) was also used to detect NanoLuc during live imaging at the single cell level using the LV200 system. Here, the diluted Nano‐Glo Live Cell Substrate solution including furimazine with dilution buffer as a 20‐fold dilution was added to the culture dish at a final dilution of 400‐fold. For real‐time monitoring of Bip promoter activity, U251/Luc stable cells were first seeded onto a glass‐bottomed dish. Real‐time monitoring of bioluminescence at the single cell level was performed in the presence or absence of chemical chaperones after the addition of d‐luciferin. CD63NLuc/U251 stable cells were also seeded onto a glass‐bottomed dish and EVs, including exosomes, were visualized after the replacement of cell culture medium with medium without FBS and the addition of a prepared substrate solution including furimazine using the Nano‐Glo Live Cell Assay System (Promega), according to the manufacturer's protocol. For time‐course analysis of CD63NLuc/U251 cells by bioluminescence imaging, hard disc (HD) recording, in which bioluminescence images were captured with every 3–5 sec exposure and recorded directly to HD by LV200 system, was performed for the observation of EV secretion from cancer cells. All data analysis was performed using AQUACOSMOS ver. 2.6 software (Hamamatsu Photonics, Shizuoka, Japan).

### 2.4 Statistical analysis

Data are expressed as the mean ± standard deviation (SD) of triplicate determinations, and statistical analysis was performed by Dunnett's test for multivalent analysis. *P*‐values less than 0.05 were considered statistically significant.

## 3 RESULTS AND DISCUSSION

### 3.1 Evaluation of chemical chaperones using real time monitoring at the single cell level

**Figure 1:**
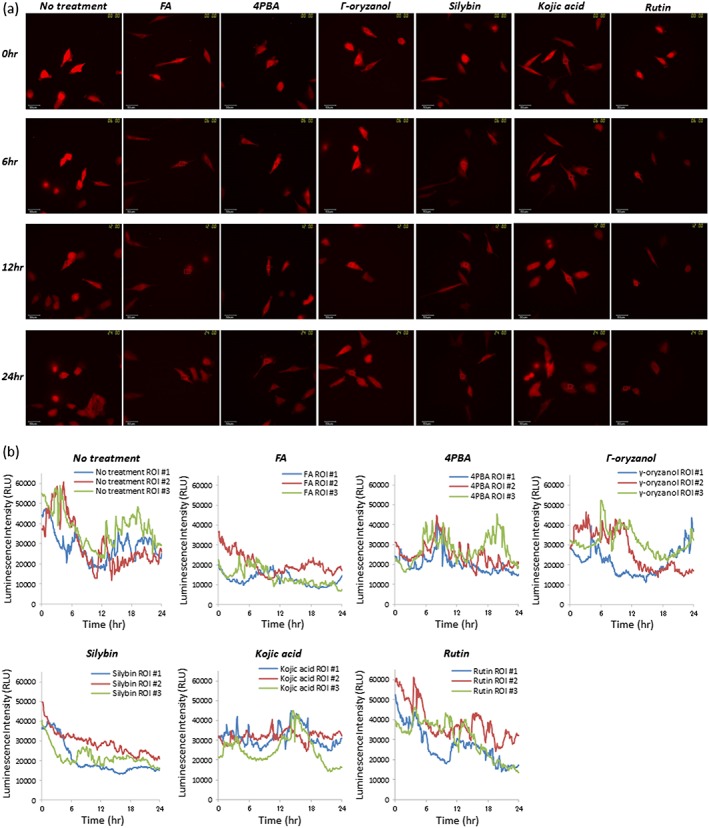
Effects of chemical chaperones on Bip promoter activation during cancer cell growth by imaging at the single cell level using bioluminescence. (a) imaging was performed using U251/Luc stable cells in the presence or absence of FA (1.5 mM), 4‐PBA (3 mM), γ‐oryzanol (20 μM), silybin (100 μM), kojic acid (1.5 mM), or rutin (100 μM) for 24 h (upper images in each panel). All bioluminescence images were captured by the LV200 system with a 120 sec exposure and a × 100 magnification oil lens after the addition of d‐luciferin, shown in red. All scale bars represent 50 μm. ROI was selected from the bioluminescence images, and average bioluminescence intensity was measured for time‐course analysis. (b) time‐course analysis of Bip promoter activity in the presence or absence of chemical chaperones at the single cell level. ROIs (#1–3) represent three independent experiments and representative images are shown in each treatment

Glioblastoma is a lethal type of malignant primary brain tumour in adults. Complete resection is currently difficult, although therapeutic approaches such as surgical treatment and chemotherapy are improving.^23^ It has been previously reported that Bip/Grp78 is a novel candidate target for improving chemosensitivity against malignant glioma, and Bip suppression in glioblastoma cells would be a novel approach to improve treatment.^24^ We first evaluated the effects of several chemical chaperones on Bip promoter activity in glioblastoma cells by bioluminescence imaging at the single cell level. As shown in Figure 1, the Bip promoter was activated during cancer cell growth, with activation peaks observed several times during long‐term observation of cancer cell growth, as reported previously.^18^ When we performed real‐time monitoring of Bip promoter activity by bioluminescence imaging at the single cell level in the presence of FA, 4PBA, γ‐oryzanol, silybin, kojic acid, or rutin, Bip promoter activity was affected and several patterns of promoter activity were observed with the chemical chaperones (Figure 1b). Activation peaks for the promoter were not observed in the presence of FA or silybin, but were still found in the presence of 4PBA, γ‐oryzanol, kojic acid, and rutin, although these peaks were lower than in those cells with no treatment (Figure 1b). It is suggested that all these compounds could affect Bip promoter activation to varying degrees during cancer cell growth. FA, silybin, and rutin also caused sustained and gradual decrease of bioluminescence intensity during monitoring. When we examined cell viability in the presence of these chemical chaperones, no compound affected cell viability dramatically (Figure S1a). Thus it is suggested that this decrease in bioluminescence intensity was not caused by damage, such as by cell death or cytotoxicity, of these compounds when analysed together with imaging in these cells using the bioluminescence microscopy system, in which marked morphological abnormalities were not observed. When the reporter assay was performed in the presence of these chemical chaperones using a luminometer, FA, silybin, and kojic acid caused a decrease in fold activation of the Bip promoter, however this decrease was slight and a marked change in fold activation was not observed using the reporter assay (Figure S1b). The luminescence intensities of selected regions of interests (ROI) from bioluminescence images at 24 h after treatment with these chemical chaperones were also examined, and a similarity with the results from the reporter assay was observed (Figure S1c). The data obtained from the reporter assay using a luminometer represent the sum of the bioluminescence from all extracted cells in the dish or well on a plate and this method is an end‐point assay. If the remaining luciferase is still active in the cells after cells had died, bioluminescence intensity is detected from the luciferase. In addition, if cells were slightly increased by treatment with chemical chaperones, overall bioluminescence intensity will be increased slightly. Thus, it is difficult to clearly discriminate bioluminescence derived from live cells or dead cells, or from slightly increased cells. However, this method is a quick and handy tool for determining the overall trend of all cells cultured in a well. Conversely it is possible to select a live cell and to exclude false positives in bioluminescence imaging using a bioluminescence microscope; this method is suitable for observing real‐time and detailed action of the promoter in a living cell. The slight difference between bioluminescence imaging and reporter assay findings might be down to the different assay methods. Real‐time monitoring of bioluminescence imaging, however, still adds to information on promoter action in living cells when combined with the conventional and classical methods. In this case, slight differences were observed as fold activation of bioluminescence intensity as described above. This bioluminescence evaluation method would allow the simultaneous visualization of cancer cell morphology and could be applied to new types of drug screening, discovery, and development.

### 3.2 Imaging of EVs including exosomes using bioluminescence

**Figure 2:**
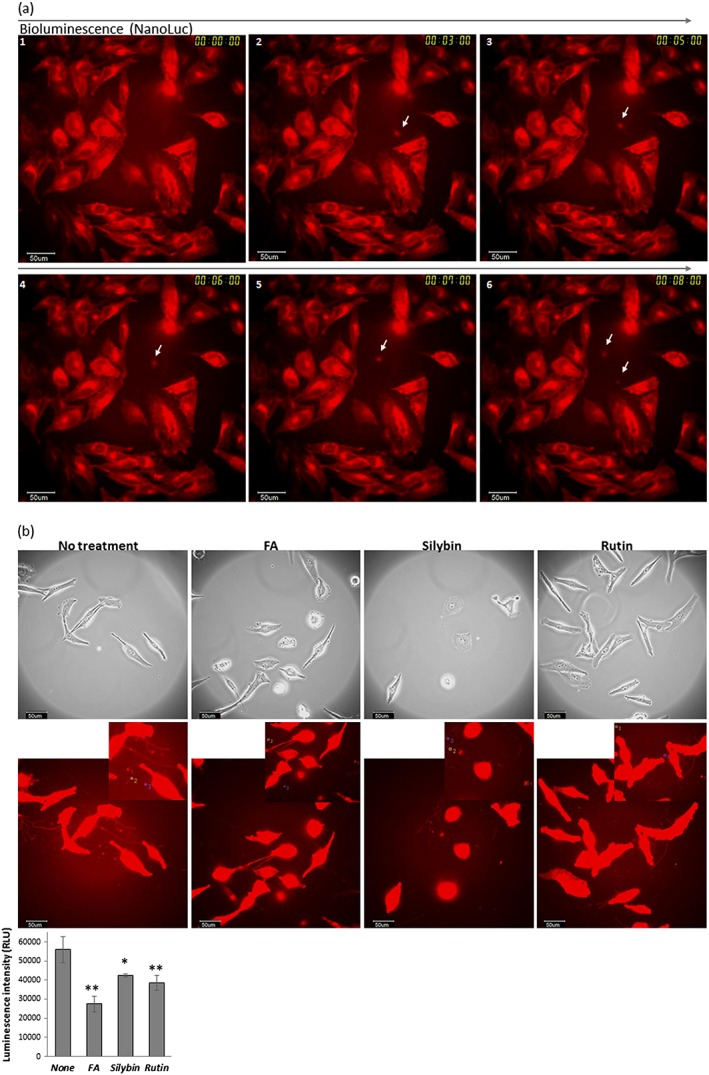
Visualization and quantification of EVs by imaging at the single cell level using bioluminescence. (a) images of CD63NLuc/U251 stable cells obtained by the LV200 system. Bioluminescence images at the single cell level were captured with a 3 sec exposure and a × 100 magnification oil lens by HD recording after the addition of substrate solution as described in the Experimental section. Numbers (1–6) and arrows in the images indicate the time course (from 0 to 10 min) during real‐time bioluminescence imaging and secreted EVs containing CD63NLuc from CD63NLuc/U251 cells, respectively. (b) bioluminescence images of CD63NLuc/U251 cells (upper images) and luminescence intensity of selected ROIs for EVs from these cells (lower graph) in the presence or absence of chemical chaperones. CD63NLuc/U251 cells were cultured on a glass plate in the presence or absence of FA (1.5 mM), silybin (100 μM), and rutin (100 μM) for 24 h, and bioluminescence images shown in red were captured with a 30 sec exposure and a × 100 oil lens after addition of substrate solution. Three ROIs were selected from the bioluminescence images, the bioluminescence intensity was measured from each ROI, and then three independent experiments were performed. Data represent the mean ± SD values from three independent experiments (total nine ROIs). (*P < 0.05, **P < 0.01 for control). Upper and lower images are phase contrast and bioluminescence images, respectively, and insets in each bioluminescence image are the magnified images with ROIs. All scale bars in the images represent 50 μm

We next visualized EVs including exosomes from cancer cells using bioluminescence imaging at the single cell level. Extracellular vesicles are currently widely investigated and receiving increasing attention in many research areas, but there are few established methods for imaging EVs using bioluminescence, although imaging using fluorescence such as with green fluorescent protein (GFP) is currently performed.^25^ We constructed an expression vector containing CD63 fused with Nano Luc luciferase (CD63NLuc) (Figure S2a), in which CD63 is a tetraspanin that is synthesized in the ER, and is required for biogenesis of a subpopulation of exosomes. CD63 is one of the most characterized marker proteins in exosomes,^13^, ^26^, ^27^ and Nano Luc is small luciferase (~19.1 kDa) derived from Oplophorus gracilirostris that can emit much higher bioluminescence intensities in the absence of ATP than firefly luciferase.^21^ We therefore established a CD63NLuc/U251 stable cell line. The expression levels of CD63 in CD63NLuc/U251 cells were measured using flow cytometry analysis and compared with those of Mock/U251 cells. It was confirmed that CD63 expression increased in CD63NLuc/U251 cells (Figure S2b), and also that EVs, including exosomes purified from the cell culture supernatant of CD63NLuc/U251 cells had sufficient bioluminescence intensity for imaging (Figure S2c). The average particle size of EVs purified from CD63NLuc/U251 cells using a capture method against phosphatidylserine on the membrane surface was 67.7 ± 22.8 nm; round vesicles were observed in transmission electron microscopy (TEM) imaging of purified EVs (Figure S2d). Bioluminescence imaging of the EVs secreted from CD63NLuc/U251 cancer cells was carried out. As shown in Figure 2a, EVs secreted from cancer cells were observed at the single cell level with the HD recording method. Real‐time imaging could be conducted for at least for 30 min with sufficient bioluminescence intensity to observe EVs when captured every 3–5 sec exposure; the overall bioluminescence intensity emitted from Nano Luc decreased during the observation period (data not shown). The use of Nano Luc might be not suitable for longer time‐course observations (such as 24 h) of live cells, as the substrate (furimazine) for the Nano Luc reaction is not as stable in cell culture medium as luciferin from firefly luciferase. Real‐time bioluminescence imaging monitoring using purified EVs and CD63NLuc at the single cell level with longer 120 sec exposures was performed. This method allowed a longer time course compared with imaging EVs secreted from cancer cells, as mentioned above (Figure S3). We could also observe and visualize the behaviour of EV including CD63NLuc close to cancer cells and performed time‐course analysis using ROIs, although the bioluminescence intensity of EV also decreased and it was not clear in this observation whether the observed EVs were taken into the cancer cells or were attached to the cell surface (Figure S3). Thus, a HD recording method with a shorter exposure and a capture method with a longer exposure would be useful for observation and visualization of secreted EVs, including exosomes from cells, to access EVs around cells or on the cell surface. These bioluminescence methods could be used as a tool to obtain new information about EVs including exosomes.

Many membranes, receptors, and secretory proteins are synthesized in the ER,^28^ the ER stress response can affect ER secretion or contents^16^, ^17^ and FA and rutin can affect and regulate ER stress.^5^, ^9^ Therefore we investigated the effects of chemical chaperones on EVs from cancer cells by bioluminescence imaging combined with effects on Bip promoter activation during cancer cell growth, in which FA and silybin affects activity dramatically and rutin causes a decrease in bioluminescence intensity (Figure 1). We examined the effects of FA, silybin, and rutin on EVs from cancer cells by reporter assay using a luminometer, FA caused a decrease in bioluminescence intensity both in the supernatant of the cell culture medium and in purified EVs; rutin also caused a slight decrease in intensity (Figure S4). These findings suggest that FA and rutin could affect EV secretion or levels of CD63NLuc expression in EVs secreted from cancer cells. We next performed bioluminescence imaging of EVs from cancer cells at the single cell level after treatment with FA, silybin, and rutin. These chaperones decreased bioluminescence intensity in the selected cancer cell EV ROIs to a greater or lesser extent (Figure 2b). These findings suggest that these chemical chaperones affected both Bip promoter activity during cancer cell growth and CD63 expression levels in EVs secreted from cancer cells, as they did not significantly affect the reaction (as enzyme–substrate) of both firefly and Nano Luc luciferase (data not shown). Silybin caused a decrease in bioluminescence intensity at the single cell level, but intensity was slightly increased in the reporter assay using a luminometer (Figures 2b and S4). Difference in these results might come from the sum of bioluminescence in the plate well for the reporter assay or from a slight increase in the number of EVs secreted from cancer cells.

EV subtypes in cancer cell lines, in which the protein expression patterns and levels were distinct, have been reported previously.^29^ We simultaneously observed both Nano Luc (EVs) and firefly Luc (fLuc) (Bip promoter activity). A CD63NLuc/BipfLuc/U251 stable cell line, in which pBipPro‐fLuc was transfected into CD63NLuc/U251 cells, was successfully established. Simultaneous observation was performed using two kinds of filters and the LV200 system to discriminate the two types of bioluminescence (Nano Luc and fLuc), These bioluminescence intensities were lower than that of individual observations through the filters (Figure S5a). In time‐lapse imaging of both Nano Luc and fLuc, the bioluminescence intensity of Nano Luc quickly decreased during the imaging (data not shown), therefore real‐time monitoring of both Nano Luc and fLuc simultaneously was not performed. However, the intensities of both EVs (Nano Luc) and Bip promoter activity (fLuc) through ROIs selection after FA treatment decreased (Figure S5b). Simultaneous observation of two types of bioluminescence at the single cell level may be a new available method, although there are currently several limitations to its use. Use of the techniques and methods described here would produce additional information when taken together with conventional and classical methods.

## 4 CONCLUSIONS

The data presented in this study describe imaging at the single cell level using bioluminescence and a new type of luciferase. Nano Luc can be used for the evaluation of chemical chaperones and also the visualization of cancer cell EVs and the EV protein expression levels. Bioluminescence imaging through the simultaneous observation of Nano Luc and fLuc was demonstrated, although there were several limitations to the described current method. These bioluminescence imaging methods could be used in the future for further studies of not only for EVs including exosomes but also for screening, discovery, and development of new compounds for cancer treatment.

## CONFLICT OF INTEREST

Ryutaro Akiyoshi, Yoko Hatta‐Ohashi, and Hirobumi Suzuki are employees of Olympus Co. Koji Kawakami serves as scientific advisor to Olympus Co. The other authors have no conflict of interest.

## Supporting information


**Figure S1** Cell viability (a), reporter (b) assays, and luminescence intensity of selected ROIs by bioluminescence imaging (c) in the presence of the chemical chaperones. U251/Luc stable cells were cultured in the presence or absence of FA (1.5 mM), 4‐PBA (3 mM), γ‐oryzanol (20 μM), silybin (100 μM), kojic acid (1.5 mM), and rutin (100 μM) for 24 h, and a cell viability (a) or reporter assay (b) was performed by the WST‐8 reagent, or using a luminometer and evaluated as fold activation for bioluminescence intensity, in which control (no treatment) was set as 1.0 as described in Experimental S1. All data represent the mean ± standard deviation (SD) from three independent experiments and each was performed in triplicate on a 96‐well plate. (c) U251/Luc cells were cultured on a glass plate in the presence or absence of chemical chaperones, and bioluminescence images at 24 h after the treatment were captured. Ten ROIs were selected from the bioluminescence images performed in three independent experiments, and the bioluminescence intensity was measured from each ROI. Data represent the mean ± SD values from 10 ROIs. (**P < 0.01 for control)
**Figure S2** Construction of expression vectors for human CD63 fused with Nano Luc reporter protein. (a) Diagrams of the domain structure of CD63 (upper image) and CD63‐NanoLuc (CD63NLuc) (lower image). The labels CytD and TMD indicate the cytosolic and transmembrane domains, respectively. The stop codon in the cDNA of CD63 was replaced with GGC for glycine, and then cloned into the multiple‐cloning site of the pNLF1C vector as described in the Experimental S1. (b) Mock/U251 or CD63NLuc/U251 stable cells were treated with a anti‐human CD63‐PE antibody, and then fluorescence activated cell sorter (FACS) analysis was performed. (c) Purified EVs from CD63NLuc/U251 cells were added to phosphate‐buffered saline (PBS) on a glass‐bottomed plate, and then the intensity of bioluminescence was examined using the LV200 system. Bioluminescence images were captured with a 30 sec exposure and a × 100 magnification oil lens after the addition of the substrate solution, shown in gray. Scale bar is 50 μm. (d) TEM image of a purified EV. Scale bar represents 100 nm
**Figure S3** Time‐lapse imaging of purified exosomes including CD63NLuc using bioluminescence. U251 cells were seeded onto a glass‐bottomed plate, purified exosomes from U251 cells transfected with CD63/pNLF1C were added to the plate, and time‐lapse imaging at the single cell level was performed by LV200 system. Images were captured with a 120 sec exposure every 5 min and a × 100 magnification oil lens after the addition of the substrate solution, shown in red (upper images). All scale bars represent 50 μm. The arrows in the images indicate exosomes containing CD63NLuc. ROIs were selected from the bioluminescence images, and average bioluminescence intensity was measured for time‐course analysis (lower graph)
**Figure S4** Effects of the chemical chaperones on EVs from cancer cells by reporter assay. CD63NLuc/U251 stable cells were cultured in the presence or absence of FA (1.5 mM), silybin (100 μM), and rutin (100 μM) for 24 h, and then a reporter assay was performed using a luminometer and evaluated as fold activation for bioluminescence intensity, in which control (no treatment) was set as 1.0, as described in Experimental S1. The labels ‘Sup’ and ‘purified exosomes’ indicate the supernatant from CD63NLuc/U251 cells cultured after treatment with or without chemical chaperones and purified exosomes from the supernatant, respectively. All data represent mean ± standard deviation (SD) values from three independent experiments and each was performed in triplicate on a 96‐well plate. (*P < 0.05, **P < 0.01 for the control)
Figure S5. Simultaneous observation of Nano Luc and fLuc at the single cell level. (a) Establishment of CD63NLuc/BipfLuc/U251 stable cells and simultaneous observation images of Nano Luc and fLuc using the LV200 system. Two types of bioluminescence (Nano Luc and fLuc) shown in blue and yellow, respectively were discriminated by two filters after the addition of two substrates described in Experimental S1. (b) Bioluminescence images of CD63NLuc/BipfLuc/U251 cells (left images) and luminescence intensity of selected ROIs for EVs (Nano Luc) and the cells (fLuc) (right graph) in the presence or absence of chemical chaperone. CD63NLuc/BipfLuc/U251 cells were cultured on a glass plate in the presence or absence of FA (1.5 mM) for 24 h, and bioluminescence images shown in blue and yellow were captured for 60 sec for Nano Luc and 120 sec for fLuc exposure, respectively and a × 100 magnification oil lens after the addition of the two substrates. Three ROIs for EVs (Nano Luc) and the cells (fLuc), respectively were selected from bioluminescence images, the bioluminescence intensity was measured from each ROI, and then three independent experiments were performed. Data represent the mean ± standard deviation (SD) from three independent experiments (total nine ROIs for Nano Luc and fLuc, respectively). (*P < 0.05, **P < 0.01 for control). All scale bars in the images represent 50 μm
