# Supplementary figures and images for: Evaluation of chemical chaperones based on the monitoring of Bip promoter activity and visualization of extracellular vesicles by real‐time bioluminescence imaging
Source: Luminescence. 2017 Sep 20;33(1):249–55. doi: 10.1002/bio.3388 (PMC6084373; doi:10.1002/bio.3388)

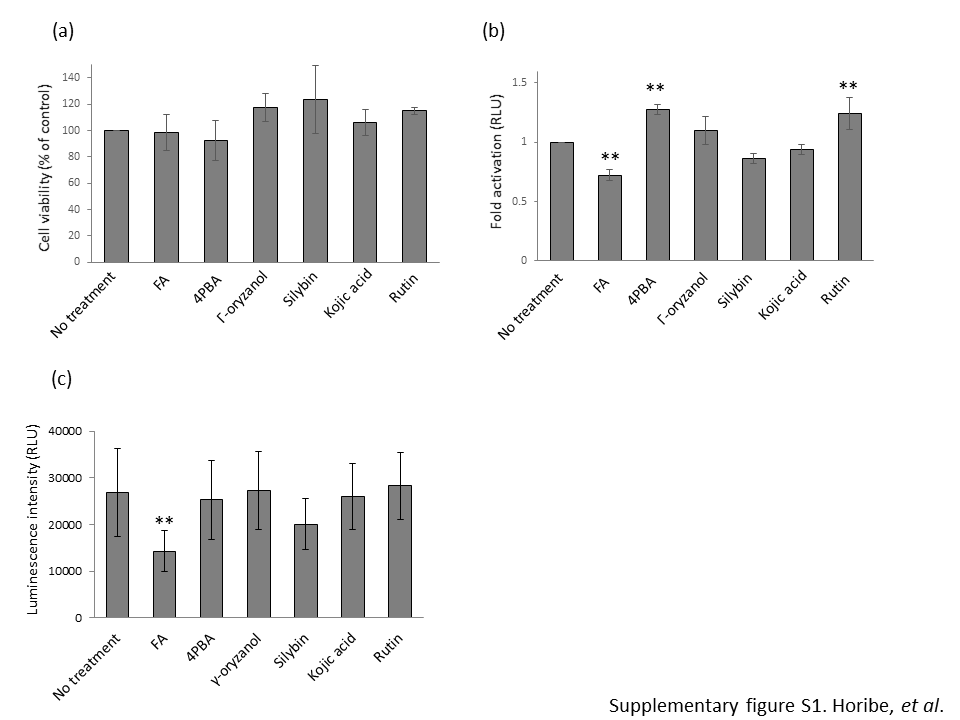

Supplement: Supplementary file 1 — Figure S1 Cell viability (a), reporter (b) assays, and luminescence intensity of selected ROIs by bioluminescence imaging (c) in the presence of the chemical chaperones. U251/Luc stable cells were cultured in the presence or absence of FA (1.5 mM), 4‐PBA (3 mM), γ‐oryzanol (20 μM), silybin (100 μM), kojic acid (1.5 mM), and rutin (100 μM) for 24 h, and a cell viability (a) or reporter assay (b) was performed by the WST‐8 reagent, or using a luminometer and evaluated as fold activation for bioluminescence intensity, in which control (no treatment) was set as 1.0 as described in Experimental S1. All data represent the mean ± standard deviation (SD) from three independent experiments and each was performed in triplicate on a 96‐well plate. (c) U251/Luc cells were cultured on a glass plate in the presence or absence of chemical chaperones, and bioluminescence images at 24 h after the treatment were captured. Ten ROIs were selected from the bioluminescence images performed in three independent experiments, and the bioluminescence intensity was measured from each ROI. Data represent the mean ± SD values from 10 ROIs. (**P < 0.01 for control) Figure S2 Construction of expression vectors for human CD63 fused with Nano Luc reporter protein. (a) Diagrams of the domain structure of CD63 (upper image) and CD63‐NanoLuc (CD63NLuc) (lower image). The labels CytD and TMD indicate the cytosolic and transmembrane domains, respectively. The stop codon in the cDNA of CD63 was replaced with GGC for glycine, and then cloned into the multiple‐cloning site of the pNLF1C vector as described in the Experimental S1. (b) Mock/U251 or CD63NLuc/U251 stable cells were treated with a anti‐human CD63‐PE antibody, and then fluorescence activated cell sorter (FACS) analysis was performed. (c) Purified EVs from CD63NLuc/U251 cells were added to phosphate‐buffered saline (PBS) on a glass‐bottomed plate, and then the intensity of bioluminescence was examined using the LV200 system. Bioluminescence image [file BIO-33-249-s001.zip › Supplementary figure S1.TIF]

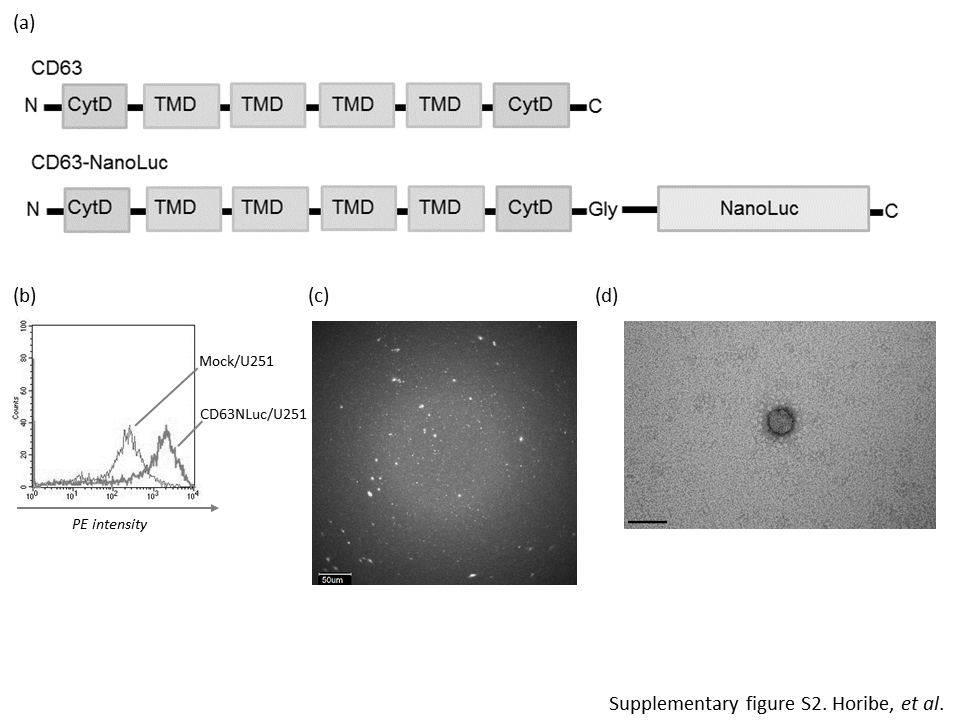

Supplement: Supplementary file 1 — Figure S1 Cell viability (a), reporter (b) assays, and luminescence intensity of selected ROIs by bioluminescence imaging (c) in the presence of the chemical chaperones. U251/Luc stable cells were cultured in the presence or absence of FA (1.5 mM), 4‐PBA (3 mM), γ‐oryzanol (20 μM), silybin (100 μM), kojic acid (1.5 mM), and rutin (100 μM) for 24 h, and a cell viability (a) or reporter assay (b) was performed by the WST‐8 reagent, or using a luminometer and evaluated as fold activation for bioluminescence intensity, in which control (no treatment) was set as 1.0 as described in Experimental S1. All data represent the mean ± standard deviation (SD) from three independent experiments and each was performed in triplicate on a 96‐well plate. (c) U251/Luc cells were cultured on a glass plate in the presence or absence of chemical chaperones, and bioluminescence images at 24 h after the treatment were captured. Ten ROIs were selected from the bioluminescence images performed in three independent experiments, and the bioluminescence intensity was measured from each ROI. Data represent the mean ± SD values from 10 ROIs. (**P < 0.01 for control) Figure S2 Construction of expression vectors for human CD63 fused with Nano Luc reporter protein. (a) Diagrams of the domain structure of CD63 (upper image) and CD63‐NanoLuc (CD63NLuc) (lower image). The labels CytD and TMD indicate the cytosolic and transmembrane domains, respectively. The stop codon in the cDNA of CD63 was replaced with GGC for glycine, and then cloned into the multiple‐cloning site of the pNLF1C vector as described in the Experimental S1. (b) Mock/U251 or CD63NLuc/U251 stable cells were treated with a anti‐human CD63‐PE antibody, and then fluorescence activated cell sorter (FACS) analysis was performed. (c) Purified EVs from CD63NLuc/U251 cells were added to phosphate‐buffered saline (PBS) on a glass‐bottomed plate, and then the intensity of bioluminescence was examined using the LV200 system. Bioluminescence image [file BIO-33-249-s001.zip › Supplementary figure S2.TIF]

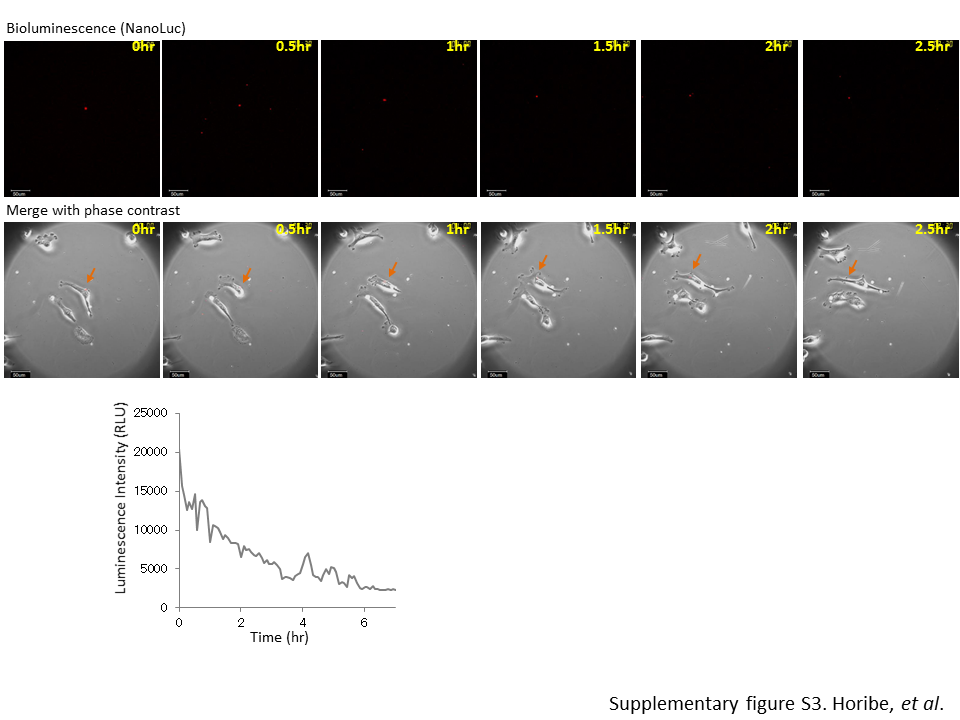

Supplement: Supplementary file 1 — Figure S1 Cell viability (a), reporter (b) assays, and luminescence intensity of selected ROIs by bioluminescence imaging (c) in the presence of the chemical chaperones. U251/Luc stable cells were cultured in the presence or absence of FA (1.5 mM), 4‐PBA (3 mM), γ‐oryzanol (20 μM), silybin (100 μM), kojic acid (1.5 mM), and rutin (100 μM) for 24 h, and a cell viability (a) or reporter assay (b) was performed by the WST‐8 reagent, or using a luminometer and evaluated as fold activation for bioluminescence intensity, in which control (no treatment) was set as 1.0 as described in Experimental S1. All data represent the mean ± standard deviation (SD) from three independent experiments and each was performed in triplicate on a 96‐well plate. (c) U251/Luc cells were cultured on a glass plate in the presence or absence of chemical chaperones, and bioluminescence images at 24 h after the treatment were captured. Ten ROIs were selected from the bioluminescence images performed in three independent experiments, and the bioluminescence intensity was measured from each ROI. Data represent the mean ± SD values from 10 ROIs. (**P < 0.01 for control) Figure S2 Construction of expression vectors for human CD63 fused with Nano Luc reporter protein. (a) Diagrams of the domain structure of CD63 (upper image) and CD63‐NanoLuc (CD63NLuc) (lower image). The labels CytD and TMD indicate the cytosolic and transmembrane domains, respectively. The stop codon in the cDNA of CD63 was replaced with GGC for glycine, and then cloned into the multiple‐cloning site of the pNLF1C vector as described in the Experimental S1. (b) Mock/U251 or CD63NLuc/U251 stable cells were treated with a anti‐human CD63‐PE antibody, and then fluorescence activated cell sorter (FACS) analysis was performed. (c) Purified EVs from CD63NLuc/U251 cells were added to phosphate‐buffered saline (PBS) on a glass‐bottomed plate, and then the intensity of bioluminescence was examined using the LV200 system. Bioluminescence image [file BIO-33-249-s001.zip › Supplementary figure S3.TIF]

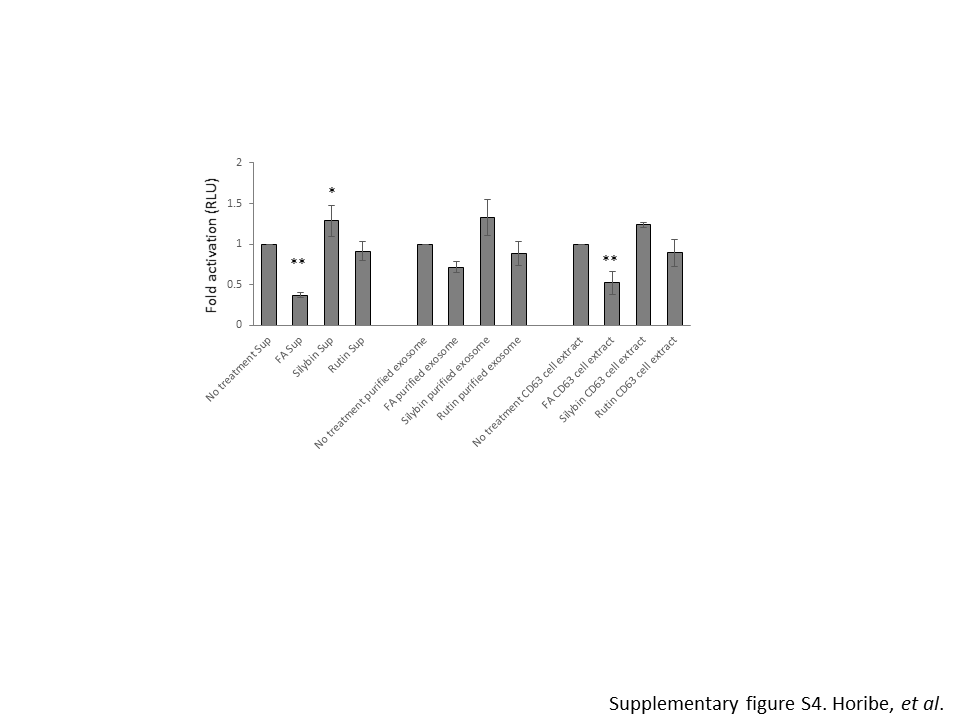

Supplement: Supplementary file 1 — Figure S1 Cell viability (a), reporter (b) assays, and luminescence intensity of selected ROIs by bioluminescence imaging (c) in the presence of the chemical chaperones. U251/Luc stable cells were cultured in the presence or absence of FA (1.5 mM), 4‐PBA (3 mM), γ‐oryzanol (20 μM), silybin (100 μM), kojic acid (1.5 mM), and rutin (100 μM) for 24 h, and a cell viability (a) or reporter assay (b) was performed by the WST‐8 reagent, or using a luminometer and evaluated as fold activation for bioluminescence intensity, in which control (no treatment) was set as 1.0 as described in Experimental S1. All data represent the mean ± standard deviation (SD) from three independent experiments and each was performed in triplicate on a 96‐well plate. (c) U251/Luc cells were cultured on a glass plate in the presence or absence of chemical chaperones, and bioluminescence images at 24 h after the treatment were captured. Ten ROIs were selected from the bioluminescence images performed in three independent experiments, and the bioluminescence intensity was measured from each ROI. Data represent the mean ± SD values from 10 ROIs. (**P < 0.01 for control) Figure S2 Construction of expression vectors for human CD63 fused with Nano Luc reporter protein. (a) Diagrams of the domain structure of CD63 (upper image) and CD63‐NanoLuc (CD63NLuc) (lower image). The labels CytD and TMD indicate the cytosolic and transmembrane domains, respectively. The stop codon in the cDNA of CD63 was replaced with GGC for glycine, and then cloned into the multiple‐cloning site of the pNLF1C vector as described in the Experimental S1. (b) Mock/U251 or CD63NLuc/U251 stable cells were treated with a anti‐human CD63‐PE antibody, and then fluorescence activated cell sorter (FACS) analysis was performed. (c) Purified EVs from CD63NLuc/U251 cells were added to phosphate‐buffered saline (PBS) on a glass‐bottomed plate, and then the intensity of bioluminescence was examined using the LV200 system. Bioluminescence image [file BIO-33-249-s001.zip › Supplementary figure S4.TIF]

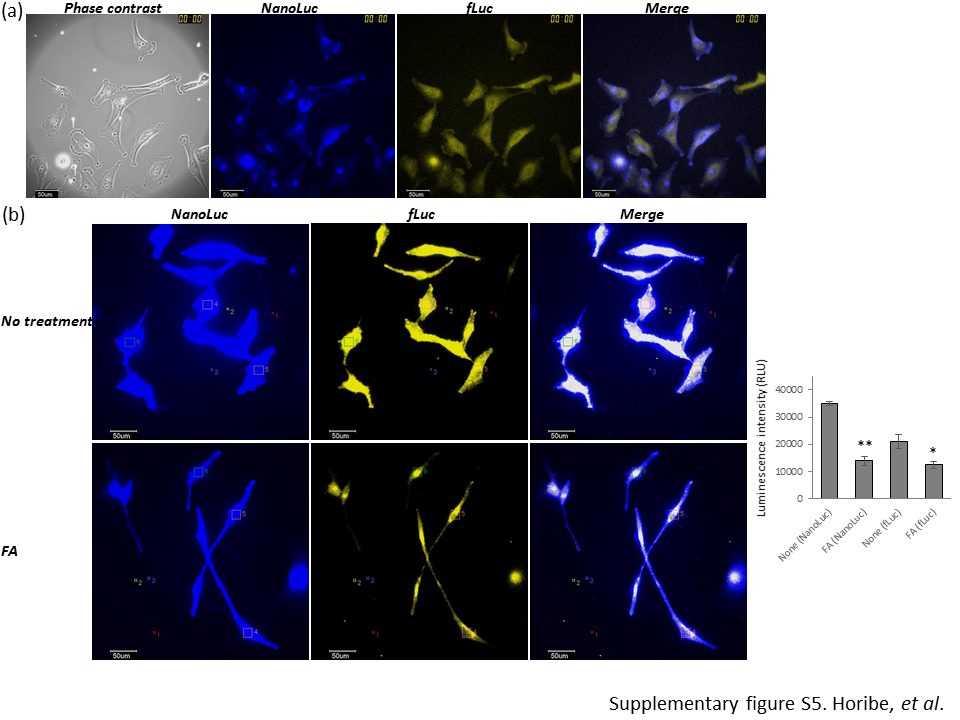

Supplement: Supplementary file 1 — Figure S1 Cell viability (a), reporter (b) assays, and luminescence intensity of selected ROIs by bioluminescence imaging (c) in the presence of the chemical chaperones. U251/Luc stable cells were cultured in the presence or absence of FA (1.5 mM), 4‐PBA (3 mM), γ‐oryzanol (20 μM), silybin (100 μM), kojic acid (1.5 mM), and rutin (100 μM) for 24 h, and a cell viability (a) or reporter assay (b) was performed by the WST‐8 reagent, or using a luminometer and evaluated as fold activation for bioluminescence intensity, in which control (no treatment) was set as 1.0 as described in Experimental S1. All data represent the mean ± standard deviation (SD) from three independent experiments and each was performed in triplicate on a 96‐well plate. (c) U251/Luc cells were cultured on a glass plate in the presence or absence of chemical chaperones, and bioluminescence images at 24 h after the treatment were captured. Ten ROIs were selected from the bioluminescence images performed in three independent experiments, and the bioluminescence intensity was measured from each ROI. Data represent the mean ± SD values from 10 ROIs. (**P < 0.01 for control) Figure S2 Construction of expression vectors for human CD63 fused with Nano Luc reporter protein. (a) Diagrams of the domain structure of CD63 (upper image) and CD63‐NanoLuc (CD63NLuc) (lower image). The labels CytD and TMD indicate the cytosolic and transmembrane domains, respectively. The stop codon in the cDNA of CD63 was replaced with GGC for glycine, and then cloned into the multiple‐cloning site of the pNLF1C vector as described in the Experimental S1. (b) Mock/U251 or CD63NLuc/U251 stable cells were treated with a anti‐human CD63‐PE antibody, and then fluorescence activated cell sorter (FACS) analysis was performed. (c) Purified EVs from CD63NLuc/U251 cells were added to phosphate‐buffered saline (PBS) on a glass‐bottomed plate, and then the intensity of bioluminescence was examined using the LV200 system. Bioluminescence image [file BIO-33-249-s001.zip › Supplementary figure S5.TIF]
